# Whole-disease-cycle management in a collaborative care model based on the WeChat platform: A case report

**DOI:** 10.1097/MD.0000000000042714

**Published:** 2025-07-25

**Authors:** Yuanyuan Fang, Libao Lu, Lingjing Teng, Pingping Qiu

**Affiliations:** a Fujian Children’s Hospital, Fujian Children’s Hospital (Fujian Branch of Shanghai Children’s Medical Center), College of Clinical Medicine for Obstetrics & Gynecology and Pediatrics, Fujian Medical University, Fuzhou Province, China; b School of Nursing, Fujian Medical University, Fuzhou Province, China.

**Keywords:** case report, congenital vaginal atresia, megacolon

## Abstract

**Rationale:**

This study aims to explore the application of a WeChat-based telemedicine collaborative care model for managing the full disease cycle of a child with Type I congenital vaginal atresia complicated by megacolon. Given the complexity of the condition, the need for multidisciplinary care, and challenges such as difficult access to follow-up, this model addresses critical gaps by providing continuous, convenient, and effective remote guidance.

**Patient concerns:**

A 13-year-old girl from the Shaxian area of Sanming City was admitted to the hospital with a diagnosis of Type I vaginal atresia and megacolon on July 9, 2023. The girl had experienced difficulty with bowel movements for the past 10 years and developed cyclical lower abdominal pain 6 months ago without any apparent cause. An abdominal and gynecological ultrasound at a local hospital revealed the absence of normal uterine, cervical, and vaginal structures.

**Diagnoses:**

In this case, we summarize the full-cycle management experience of 1 case of Type I vaginal atresia combined with megacolon.

**Interventions:**

Key nursing points include: forming a multidisciplinary collaborative care team, and implementing precise management throughout the process using a telemedicine model.

**Outcomes:**

Preoperatively, gynecologists from external hospitals were consulted to develop preoperative treatment, care plans, and surgical strategies. Intraoperatively, enhanced cooperation among surgeons, anesthesiologists, and nurses ensured the smooth progress of the surgery. Postoperatively, the multidisciplinary team provided timely, effective, convenient, and cost-efficient management through real-time remote guidance based on the patient’s condition. During home care, communication with multidisciplinary professionals was maintained via WeChat, establishing a telemedicine-based continuity of care plan.

**Lessons:**

This case highlights the effectiveness of a WeChat-based collaborative care model in the full-cycle management of a child with Type I vaginal atresia complicated by megacolon, providing convenient, professional, and continuous treatment and care with significant clinical benefits.

## 1. Introduction

Congenital vaginal atresia results from abnormal development of the urogenital sinus and the terminal ends of the Müllerian ducts, leading to an unformed or non-patent vagina. It is a relatively rare condition among female genital tract malformations, with an incidence of approximately 1 in 4000 to 1 in 10,000 cases.^[1–4]^ Type I congenital vaginal atresia is a specific congenital reproductive tract malformation characterized by a well-developed uterus combined with partial vaginal atresia.^[5]^ Surgical intervention is the only treatment for congenital vaginal atresia. Delayed treatment not only increases patient suffering but can also lead to severe pelvic endometriosis and adenomyosis due to retrograde menstruation, potentially impairing fertility.^[6]^ However, early diagnosis is challenging, surgical procedures are difficult, and there is a risk of postoperative complications such as re-adhesion, atresia, and infection.^[7]^ Additionally, long-term follow-up is required postoperatively. For patients who live far from the hospital or have difficulty visiting, regular face-to-face follow-up is often impractical, and telephone follow-up has a high rate of loss to follow-up and low intervention efficiency, leading to a lack of continuity in the patient’s care plan.^[8]^ Furthermore, this condition is often complicated by other malformations, necessitating multidisciplinary collaboration for clinical diagnosis, treatment, and care. For specialties not available in our hospital, external specialists need to be invited for guidance. Given these challenges, effective treatment and care for this condition require a multidisciplinary team capable of providing real-time guidance and management to the patient and their family.

With the rapid development of the internet, digital health interventions, accessible via smartphones, tablets, and computers, offer a promising platform for providing health-related services and implementing intervention plans effectively and conveniently.^[9]^ The advent and ongoing development of telemedicine have significantly met the demands of modern healthcare, reducing barriers to providing healthcare management. By utilizing electronic devices, sensors, and internet technologies, patient data, voice, and images can be transmitted online. Telemedicine represents a new patient-centered medical service system that integrates remote monitoring, diagnosis, treatment, and management.^[10,11]^ In recent years, the continuous nursing model of WeChat platform has been widely used in various diseases to guide the rehabilitation of patients after discharge and reduce complications, the improvement of self-care ability and quality of life has achieved remarkable results.^[12]^ The management of children with Type I vaginal atresia, from preoperative to postoperative care and long-term follow-up after discharge, requires multidisciplinary collaboration throughout the disease cycle. However, due to geographical, temporal, and economic limitations, existing medical models may have significant limitations, hindering the recovery of the child’s physical and mental health. In this case report, we propose the application of a telemedicine model based on a collaborative care team throughout the disease cycle of a child with Type I vaginal atresia, demonstrating its positive role in remote management and offering a convenient, effective, and low-cost management solution.

## 2. Patient information

A 13-year-old girl from the Shaxian area of Sanming City was admitted to the hospital with a diagnosis of Type I vaginal atresia and megacolon on July 9, 2023. The girl had experienced difficulty with bowel movements for the past 10 years and developed cyclical lower abdominal pain 6 months ago without any apparent cause. An abdominal and gynecological ultrasound at a local hospital revealed the absence of normal uterine, cervical, and vaginal structures. Due to the local hospital’s limited resources, the patient was referred to a higher-level hospital for treatment. The informed consent was given, and this study was approved by the ethics committee of Fujian Children’s Hospital (2022ETKLRK09013).

## 3. Clinical findings

Upon admission to our department, the patient underwent preoperative examinations as per medical advice and received daily retrograde enemas. Based on the gynecological consultation, she was also administered Triptorelin to suppress menstruation. During this period, the patient developed a fever, which was considered to be due to pelvic hematoma with infection. A general surgeon performed an “ultrasound-guided percutaneous cervical cavity puncture and drainage procedure.” Following detailed discussions among the multidisciplinary team, a comprehensive treatment plan was formulated, and on August 23, 2023, the patient underwent “laparoscopic exploration and transverse colostomy.” During the procedure, extensive pelvic and abdominal endometriosis with secondary acute inflammation was noted, leading to the decision to postpone the vaginal surgery. The patient continued to receive Triptorelin for menstruation suppression. Postoperatively, a consultation with the nutrition department revealed a high nutritional risk, and parenteral nutrition support was provided.

On October 13, the patient underwent “1. Vaginoplasty 2. Megacolon radical surgery,” both of which were successful. By October 23, the patient was in good general condition and was discharged with instructions to continue gynecological follow-up and to undergo anal dilation 1 week later. On May 30, 2024, she returned to the hospital for a “colostomy closure and partial transverse colon resection.” Her postoperative recovery was good, and she was discharged on June 9, 2024, with instructions to follow-up in the outpatient clinic. Follow-up to date shows that the patient is in good general condition, with normal bowel movements and menstruation, and no significant discomfort.

## 4. Discussion

In this case, the patient required multidisciplinary collaboration throughout the entire disease cycle, involving general surgery nurses, general surgeons, gynecologists, anesthesiologists, wound and ostomy care specialists, nutritionists, and psychologists. Therefore, we established a nurse-led collaborative care team. This care model centers on a well-prepared, proactive, and interdisciplinary nursing team, providing care for proactive patients and their families.^[13]^ The collaborative care model also actively encourages parents to participate in the child’s care, making them the primary caregivers in the daily management of the child’s condition. This approach helps alleviate the fear and anxiety associated with hospitalization.^[14]^ This involvement is especially crucial for tasks such as stoma care and vaginal mold maintenance during the post-colostomy home care period, which must be performed by the family members.

Research by Zhu and her colleagues has shown that patients highly appreciate the intervention model based on a WeChat platform for multidisciplinary collaborative teams.^[15]^ Since our hospital did not have an in-house gynecologist, we requested a consultation from an external gynecologist after the patient was admitted to the general surgery department. However, monitoring the patient’s condition and implementing the necessary treatment and care measures preoperatively and postoperatively required the guidance of a multidisciplinary team. Therefore, we decided to manage the patient through telemedicine, forming a WeChat group with the collaborative care team and the patient’s family, allowing everyone to participate online in the development of the treatment and care plan for the patient.

Tang et al^[16]^ have confirmed that when a stoma closure is postponed due to the patient’s condition, home care for the stoma becomes challenging, with a high risk of complications. In this case, the patient’s family managed stoma care for over 7 months after the patient was discharged following colostomy surgery. Before discharge, the ostomy care specialist created a personal care profile for the patient, provided video links for home stoma care, and explained in detail the potential stoma-related complications, including how to monitor for, manage these complications, and replace the stoma bag. The patient’s understanding of these procedures was assessed.^[17]^

Additionally, the patient required long-term placement of a vaginal mold post-surgery. After discharge, specialized team members provided targeted guidance based on the patient’s specific needs. The patient’s family could consult via the WeChat group by sending text, pictures, or videos to address any concerns. The multidisciplinary team members responded promptly online, answering questions and effectively resolving issues, thereby reducing the need for frequent hospital visits, which had both economic and social benefits. Due to the nature of this condition, the patient often experienced pain both before and after surgery. Friedrichsdorf and Goubert^18]^ suggest that multimodal analgesia is the gold standard in pain management and emphasize the importance of non-pharmacological treatments in managing pediatric pain. During the perioperative period, the responsible nurse instructed the patient on how to accurately express pain and understand the VAS (Visual Analogue Scale) pain scoring method. When the VAS score was <4, the nurse implemented mindfulness music therapy by selecting calm and soothing music that the patient liked. The patient, accompanied by a parent, was guided to focus on the bodily and sensory changes brought about by the music, which helped shift attention, induce pleasant feelings, promote relaxation, and reduce pain levels.^[19,20]^ When the VAS score was 4 or higher, the nurse posted the pain score in the WeChat group, and the anesthesiologist promptly provided guidance for pharmacological pain relief via WeChat, thereby alleviating the pain from the patients.

Moreover, since the patient was in adolescence – a psychologically sensitive period – the family was also anxious due to their lack of knowledge about the disease and concerns over its treatment and prognosis. After forming the multidisciplinary team, the psychologist provided PERMA theory training to the general surgery nurses. Throughout the disease cycle, the psychologist guided the nursing staff via the WeChat platform to use positive psychological interventions based on PERMA theory. These interventions helped the patient develop positive emotions, build relationships, receive more affirmation and encouragement, and gain spiritual support, enabling the patient to meet inner needs and face challenges with a positive attitude, ultimately improving quality of life.^[21]^ Additionally, when the patient or family experienced negative emotions, the psychologist offered timely remote counseling via WeChat, avoiding the awkwardness of face-to-face sessions and making it easier for the patient and family to open up. As described by Temkin-Greener^[22]^ and Adler Milstein et al,^[23]^ telemedicine has been widely used among dementia patients, both during and after the pandemic, and has been well-received by patients, families, and caregivers, offering significant advantages. Additionally, studies^[24]^ have shown that the application of telemedicine in pediatric intensive care units facilitates case discussions and personalized treatments tailored to the needs of patients and the resources of the institution, with team members expressing high levels of satisfaction. However, the use of telemedicine in cases of Type I vaginal atresia combined with megacolon is extremely rare.

This case report also has several limitations: the use of the WeChat platform for positive psychological interventions limited the ability to observe the patient’s facial expressions and tone of voice, which are important for assessing emotional responses; the follow-up period was relatively short, preventing an evaluation of the patient’s long-term quality of life; as this is a single case report, the conclusions drawn may be subject to unavoidable bias due to individual variability. In the future, increasing the sample size will be essential to provide more robust clinical evidence for this approach. We believe that this management model holds great potential for broader implementation and could lead to highly satisfactory outcomes.

## 5. Conclusion

Type I vaginal atresia is a rare and complex condition, and when accompanied by megacolon, its management becomes even more challenging due to the involvement of multiple specialties. In this case, a WeChat-based multidisciplinary collaborative care model was employed to provide comprehensive, full-cycle disease management, achieving favorable outcomes. Personalized treatment plans were developed by specialists from various disciplines and implemented throughout both the perioperative period and postdischarge follow-up. This approach effectively addressed limitations related to time and geographic access to care, offering a practical solution grounded in the recognition of traditional treatment and follow-up constraints.

## Author contributions

**Conceptualization:** Libao Lu, Pingping Qiu.

**Data curation:** Yuanyuan Fang.

**Funding acquisition:** Yuanyuan Fang.

**Investigation:** Libao Lu.

**Methodology:** Yuanyuan Fang, Lingjing Teng.

**Project administration:** Pingping Qiu.

**Software:** Yuanyuan Fang, Libao Lu.

**Supervision:** Libao Lu.

**Validation:** Yuanyuan Fang, Libao Lu.

**Visualization:** Libao Lu, Pingping Qiu.

**Writing – original draft:** Yuanyuan Fang, Libao Lu.

**Writing – review & editing:** Libao Lu, Lingjing Teng, Pingping Qiu.
